# Supplementary material for: Assessment of Sustainable Ethanolamine-Based Protic Ionic Liquids with Varied Carboxylic Acid Chains as Corrosion Inhibitors for Carbon Steel in Saline Environments
Source: Molecules. 2025 Feb 24;30(5):1033. doi: 10.3390/molecules30051033 (PMC11901724; doi:10.3390/molecules30051033)
Supplement: Supplementary file 1 [file molecules-30-01033-s001.zip › molecules-3463640-supplementary.pdf]

Supplementary Information

1- *Protic Ionic Liquids characterization*

A thorough physicochemical analysis was conducted to elucidate the electrochemical behavior of protic ionic liquids, as corrosion inhibitors, interacting with metallic surfaces in saline solution (NaCl 3.5 % wt.). Indeed, the properties evaluated included dynamic and kinematic viscosity, density, conductivity in NaCl solution, pH, sound velocity, refractive index, and moisture content [1].

Initially, viscosity measurements were classified into two types: dynamic and kinematic, to understand the behavior of PILs in saline electrolytes. Dynamic viscosity (μμ) refers to the force required to move a unit area of a compound over a unit distance. Kinematic viscosity, on the other hand, is derived by dividing dynamic viscosity by the density of the fluid under investigation. Both parameters are essential for understanding the kinetic interactions and synergism of the molecules studied.

Dynamic viscosity measurements revealed that, as the carbon chain length of the acids used in this study increased, there was a proportional rise in viscosity values, ranging from 23.94 to 102.4 mPa·S. Similarly, kinematic viscosity followed a parallel trend, with values increasing from 20.76 to 97.46 mm22/s. Additionally, the density of the PILs demonstrated an inverse correlation with the carbon chain length, decreasing from 1.15 to 1.05 g/cm33 [2–6]. In the electrochemical results obtained and discussed in the paper, these factors, in this case, the size of the carbon chain, were a crucial factor in the inhibitory capacity of the compounds tested.

Table S1
Protic Ionic Liquids characterization (abbreviations, structures, supplier molecular, density, viscosity, conductivity, and pH).

PILs Abbrev.	V. Dynamic (mPa.S)*	V. Kinematics (mm2/S)*	Molar Mass	Density	*Cond	*pH	Speed of sound (m/s)	Refractive Index (nD)	Moisture
PIL-1	23,940	20,760	107.1	1,1562	16,88	8,96	1806,5	1,4507	6,11%
PIL-2	43,980	39,712	135.0	1,1075	17,30	6,48	1810,1	1,4427	8,22%
PIL-3	102,400	97,464	163.1	1,0507	16,85	5,89	1615,0	1,4483	5,74%

*NaCl 3.5% pH and conductivity.

The assessment of conductivity and pH in a 3.5 % wt. NaCl solution is directly associated to the study of corrosion inhibitors. The addition of certain compounds to the solution is not aimed at altering the factors, such as pH or conductivity [1,7]. Mainly, thermodynamic characterizations of the interactions between corrosion inhibitors and electrolytes provide valuable insights into specific macroscopic properties. Notably, evaluating the density and speed of sound of various chemical components, such as Protic Ionic Liquids (PILs), offers a more comprehensive comprehension of the chemical interactions among the solution's species, aiding in corrosion prevention investigations [8].

Keshapolla et al. [8], emphasize the importance of sound waves in generating precise information about the molecular phenomena, in this case inhibitors, and steel surfaces when interacting with PILs. In this study, the speed of sound of the examined PILs was measured (Details in the Table S1). In sum, the results revealed an increase in sound velocity with the elongation of the chain length, especially in carboxylic acids compounds. Indeed, such observations are especially valuable for determining the fundamental forms of certain chemicals used in applications, such as gases capture, catalysis and corrosion prevention.

Nonetheless, the results for PILs exhibited a contrasting trend, as an increase in carbon chain length resulted in a decrease in the speed of sound. In total, this behavior is likely attributed to the structural nature of PILs, which differ from Aprotic Ionic Liquids (AILs) in both structural class and properties. Moreover, the speed of sound values obtained for the studied PILs (1615–1810 $m.s^{-1}$) were comparable to those reported by Sharma et al. for similar compounds (1415–1566 $m.s^{-1}$), reinforcing the consistency of chemical behavior and possibility of usage in various applications, in the case of the article as inhibitors, after the electrochemical and mass loss tests, the protic ionic liquids presented a reasonable (values above 80% efficiency) response to the demand in the conditions investigated with saline medium and high chloride concentration. [9].

In addition to these physical characterizations, the refractive index of PILs was also evaluated to clarify the chemical interactions within the electrolyte/steel/PIL system. The refractive index provides critical data on molecular electronic polarizability, facilitating an understanding of the interactive forces between molecules or their behavior in solution (Table S1) [3].

Indeed, Almeida et al. [10] underlined that the refractive index is affected directly by the anion's volume within the solution. In truth, in the instance of imidazolium-based ionic liquids, modifications such as the addition of functional groups or the extension of alkyl chains enhance the electron density surrounding the alkyl group, and also with this modification of the size of the carbon chain, for example, there is a totally different coating behavior on the metal surface. For the study of inhibitors, the variation of chemical formulations is an aspect that always deserves to be investigated because a good protective action of an inhibitor when modified when taken to a different electrolytic medium the behavior can be totally different, positively or even negatively [6].

Deetlefs et al. [11] argue a crucial arguments about the refractive index factor, because according then this factor correlates strongly with the molar volume's increasement of the chemical compounds. This perspective is particularly relevant for advancing studies focused on the intricate atomic interactions in ionic liquids. Nonetheless, findings related to the application of protic and aprotic ionic liquids may differ when viewed from an electrochemical standpoint. For instance, the carbon chain derived from the carboxylic acid (Anionic Fraction) can interact differently with the metallic surface, depending on whether the electrolyte environment is basic or acidic.

Therefore, in Table 1, the average refractive index found in work was approximately 1.4472 nD at 300k, while the results supported by Sandar [3] were 1.51-1.54 nD average at the same temperature. Likewise, Almeida's [10] average at 300 k was 1.44-1.48 nD, and S. Sardar [6] average was 1.49 - 1.55 nD. Thus, as all the above results show, a close average of values can confirm how important it is to carry out the physical-chemical characterization of ionic liquids, whether aprotic or protic.

In fact, from the moisture values presented in table 1, it is clear to observe that protic ionic liquids are highly hygroscopic compared to other inhibitors, which corroborates the presence of the broad signal in the 4.3 to 4.8 ppm range in the spectra of hydrogen, which is a characteristic of the water present in the sample 6.69% average. This value is nearly approximate to the water content found by Viesca in 2020, in their studies 9.2% average, where they had a minimum water content of 0,6% and a maximum of 18.1%. The successive observation is essential to explain why the water content factor is not a massive issue for this application, corrosion inhibitor [8,10,12].

2- RMN-H

Figures **S1** and **S2** indicate the ^{1}H and ^{13}C NMR spectra of PIL 01, 02, and 03. Its main structural modifications can be identified and used as evidence of the formation of the acid-base reaction due to the combination of 2-hydroxyethylamine and three different carboxylic acids (formic, propanoic, and pentatonic), as reported in the literature [1,13,14].

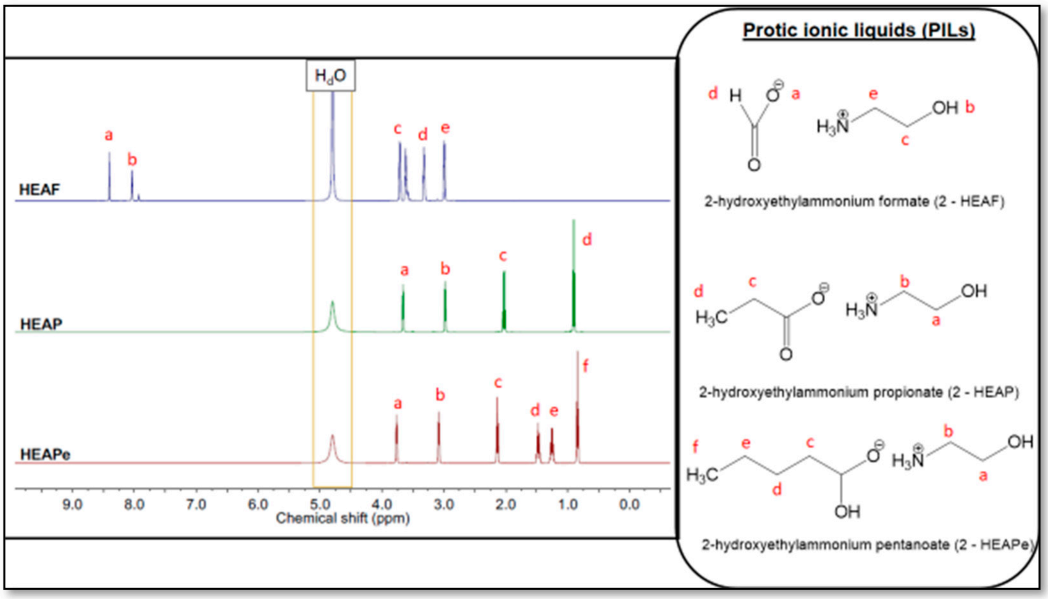

Figure S1. ^{1}H NMR RMN PIL (PIL 01 / 2-HEAF), (PIL 02 / 2-HEAP) and (PIL 03/ 2-HEAPe).

In detail, the characteristic signals observed between 8.5 and 8.0 ppm in the hydrogen spectrum **(S1)** [15] are attributed to the primary carboxyl proton. Additionally, a distinct signal consistently detected across all ^{1}H spectra within the range of 4.3 to 4.8 ppm is indicative of water originating from ambient humidity. This observation aligns with the high hygroscopic nature of PILs, which results in moisture content averaging approximately 6.5% in their composition [12,13,16].

Moreover, the ^{1}H NMR technique is indispensable for the chemical characterization and evaluation of synthesized compounds, as the spectra shown in **Figure S1** confirm the successful synthesis and validate the material to be evaluated as corrosion inhibitors in the article [3,12,13,17,18].The spectra clearly exhibit the presence of hydrogen bonded to a carboxyl group, reflecting the significant prevalence of carboxylic acids in PILs [13,17]. This prominence arises from the crucial role of these acids in the synthesis of sustainable ionic liquids, valued for their reduced toxicity and minimal impact on human health.

In this study, the acids selected—formic, propanoic, and pentanoic—share a common chemical structure, with variations introduced intentionally through differences in their carbon chain lengths. Additionally, the amine compounds are located at the extreme end of the positive segment of the PILs, positioned opposite the hydroxyl group (b).

3- RMN-C

Then, **Figure S2** confirms the ^{13}C NMR spectra of PIL 01, 02, and 03, correspondingly, in which their main structural carbon compounds can be identified and used as evidence to prove the formation of PILs.

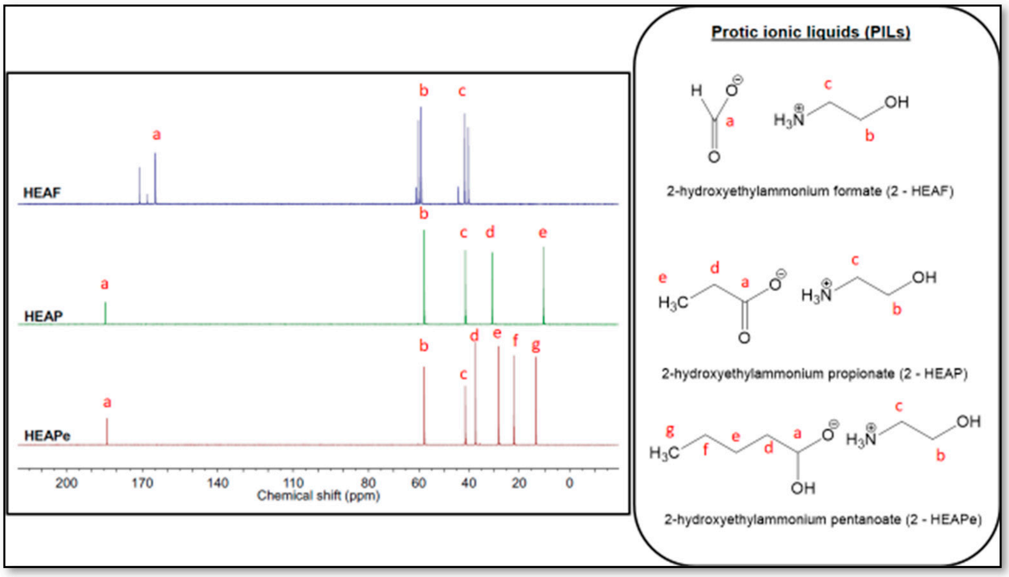

Figure S2. ^{13}C RMN spectrum of PIL (PIL 01 / 2-HEAF), (PIL 02 / 2-HEAP) and (PIL 03/ 2-HEAPe).

In the initial analysis of the ^{13}C NMR spectra (Figure S2), signals observed in the range of 170–190 ppm indicate the presence of carbonyl (C=O) groups in the protic ionic liquid molecules under evaluation. These signals are attributed to the acid-base reaction between 2-hydroxyethylamine and formic, propanoic, and pentanoic acids, confirming the successful synthesis of the intended compounds. The spectral data is consistent with the expected structural transformations.

In a previous evaluation of another region of the protic ionic liquid (PIL) complex [12], two distinct scenarios were observed. First, the carbon atom bonded to a highly electronegative hydroxyl group (OH) exhibited a signal around 60 ppm, highlighting its unprotected nature in the PIL structure.

Second, the carbon atom with a signal at approximately 40 ppm showed a comparatively minor interaction with the polar fraction of the molecule, potentially due to greater steric protection. Lastly, the signals represented by regions (d–g) correspond to the alkyl chain of the acids. As the carbon chain length increases from HEAF to HEAPe, additional carbon atoms are incorporated. Consequently, in the final spectrum, these atoms shift further to the right, reflecting their increased distance from the compound's electronegative region.

4- FTIR spectroscopy

One of the most important characterization techniques, FTIR analysis supported the assessment of structural variations among the protic ionic liquids (PILs) examined in this study. In sum, the absorption spectra of PIL 01, PIL 02, and PIL 03, as depicted in **Figure S3**, reveal distinct features indicative of their molecular compositions.

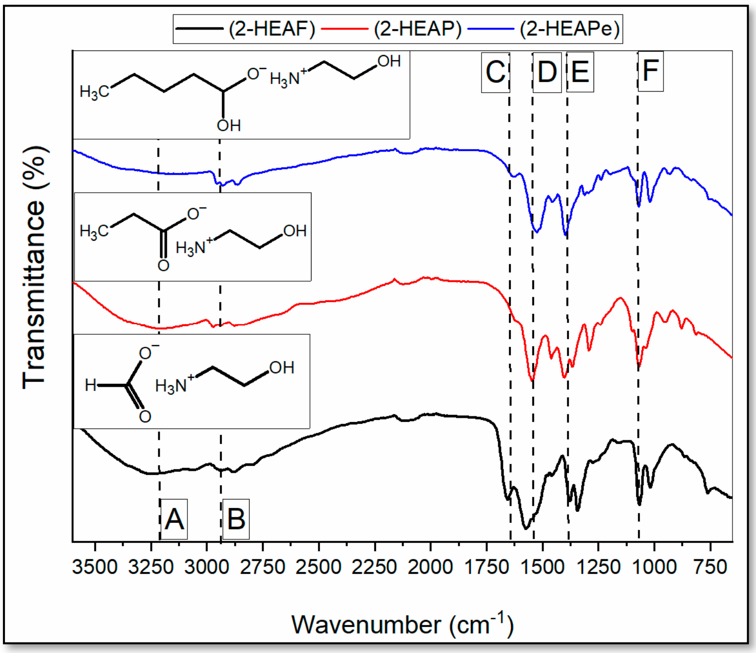

Figure S3. FT-IR spectra of (PIL 01 / 2-HEAF), (PIL 02 / 2-HEAP) and (PIL 03/ 2-HEAPe).

Notably, a broad spectral region, highlighted by sections A and B within the range of 3600-2250 cm^{-1}, showcases key vibrational modes. This region includes the O-H stretching band and the N-H stretching band, located between 3600-3200 cm^{-1}, along with the C-H stretching band observed in the range of 3000-2800 cm^{-1}. These bands are characteristic of the ammonium cation structure, reflecting the fundamental interactions within the PILs. The identification of these specific absorption bands underscores the structural integrity and chemical functionality of the PILs under evaluation [1,13,14].

Furthermore, in sections C and D, two bands are noted at (1650 cm^{-1}) and (1550 cm^{-1}). These bands can be attributed to the C=O asymmetric stretches of anionic carboxylate and neutral carboxylate groups [1,13,14]. Finally, in the regions marked as E and F, bands with (1375 cm^{-1}) are observed, which can be related to the symmetric angular deformation band CH_2 and (1090 cm^{-1}) attributed to the C-N stretch band.

5- *Thermal gravimetric analysis*

Thermogravimetric analysis (TGA) is extensively recognized as an essential technique for evaluating the thermal stability of chemical compounds. This stability is typically characterized by the onset temperature (T_{onset}), defined as the intersection point of two tangent lines drawn on the TGA curve, one preceding and the other following material decomposition [3,14].

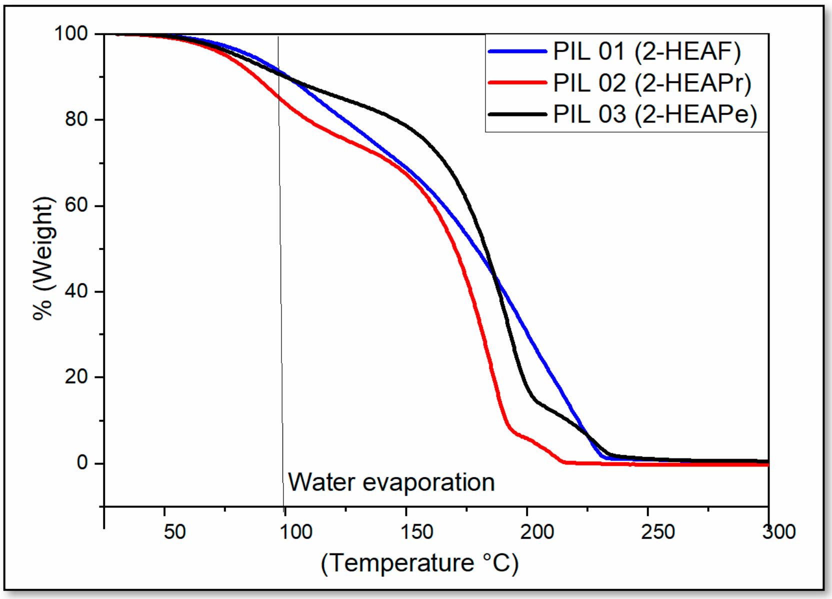

Figure. S4. Thermogravimetric analysis of the studied PILs

Figure S4 illustrates a representative thermal decomposition profile of the three protic ionic liquids (PILs). The curve reveals a distinct plateau following the evaporation of water at approximately 100 °C, with the onset temperature ranging from 160.25 °C to 171.45 °C. These inferences clearly demonstrate the considerable thermal stability of PILs, particularly as the temperature increases. This property represents a significant advantage over certain types of green corrosion inhibitors, such as organic inhibitors, which often lack substantial thermal stability. As a result, countless of these inhibitors are unsuitable for studies where thermal resistance is a critical factor.

In the context of this research, the thermal stability of PILs reinforces their potential application in environments where varying temperature conditions are present. By ensuring that their corrosion inhibition performance remains consistent across different temperatures, this study highlights the practical advantages of PILs in comparison to other inhibitors. This focus on thermal behavior further establishes a basis for understanding the broader applicability of PILs in corrosion prevention studies [13].

Table S2
Thermogravimetric temperature values obtained from PILs (PIL 01, PIL 02, and PIL 03)

PIL	Onset	Tmáx
HEAF	171,45C	198,36 C
HEAP	160,25C	180,15 C
HEAPe	170,59C	188,86 C

The impact of incorporating specific functional groups into the structure of ionic liquids on their thermal degradation has been extensively reported in the literature [19–22]. In alignment with these findings, the present study observed comparable trends. Specifically, the addition of a carbon atom to the main chain of carboxylic acid resulted in a modest reduction of the thermal degradation temperature by approximately 10°C.

This observation enhances the understanding of how the structural composition of ionic liquids influences their thermal properties. Notably, smaller ionic liquid compounds, characterized by fewer atoms, can potentially yield a more thermally stable and resilient product compared to their larger counterparts. Such a conclusion emphasizes the critical role of molecular size in determining the thermal behavior of ionic liquids.

The relationship between structural modifications and thermal stability is further illustrated by the data presented in Table S2. Here, the onset temperature and the maximum degradation temperature (Tmax) values are shown to correlate with the addition of carbon to the molecular chain. These findings provide valuable insights into the design of ionic liquids with tailored thermal properties, offering opportunities to optimize their stability for various applications. By understanding these nuances, the study contributes to the broader goal of developing ionic liquids with enhanced performance and stability under specific conditions.

REFERENCES

[1] N. Bicak, A new ionic liquid: 2-hydroxy ethylammonium formate, J. Mol. Liq. 116 (2005) 15–18. https://doi.org/10.1016/j.molliq.2004.03.006.

[2] H. Ohno, Functional design of ionic liquids, Bull. Chem. Soc. Jpn. 79 (2006) 1665–1680. https://doi.org/10.1246/bcsj.79.1665.

[3] S. Sardar, C.D. Wilfred, A. Mumtaz, J.M. Leveque, A.S. Khan, S. Krishnan, Physicochemical properties, Brönsted acidity and ecotoxicity of imidazolium-based organic salts: Non-toxic variants of protic ionic liquids, J. Mol. Liq. 269 (2018) 178–186. https://doi.org/10.1016/j.molliq.2018.08.017.

[4] M. David, Applications of ionic liquids in polymer science and technology, 2015. https://doi.org/10.1007/978-3-662-44903-5.

[5] A. Hayyan, F.S. Mjalli, I.M. Alnashef, T. Al-Wahaibi, Y.M. Al-Wahaibi, M.A. Hashim, Fruit sugar-based deep eutectic solvents and their physical properties, Thermochim. Acta. 541 (2012) 70–75. https://doi.org/10.1016/j.tca.2012.04.030.

[6] S. Sardar, C.D. Wilfred, A. Mumtaz, J.M. Leveque, Investigation of the Thermophysical Properties of AMPS-Based Aprotic Ionic Liquids for Potential Application in CO2 Sorption Processes, J. Chem. Eng. Data. 62 (2017) 4160–4168. https://doi.org/10.1021/acs.jced.7b00552.

[7] T.E. Schmitzhaus, M.R. Ortega Vega, R. Schroeder, I.L. Muller, S. Mattedi, C. de F. Malfatti, An amino-based protic ionic liquid as a corrosion inhibitor of mild steel in aqueous chloride solutions., Mater. Corros. (2020). https://doi.org/10.1002/maco.201911347.

[8] D. Keshapolla, K. Srinivasarao, R.L. Gardas, Influence of temperature and alkyl chain length on physicochemical properties of trihexyl- and trioctylammonium based protic ionic liquids, J. Chem. Thermodyn. 133 (2019) 170–180. https://doi.org/10.1016/j.jct.2019.02.015.

[9] G. Sharma, R.L. Gardas, A. Coronas, G. Venkatarathnam, Effect of anion chain length on physicochemical properties of N,N-dimethylethanolammonium based protic ionic liquids, Fluid Phase Equilib. 415 (2016) 1–7. https://doi.org/10.1016/j.fluid.2016.01.036.

[10] H.F.D. Almeida, J.A. Lopes-Da-Silva, M.G. Freire, J.A.P. Coutinho, Surface tension and refractive index of pure and water-saturated tetradecyltrihexylphosphonium-based ionic liquids, J. Chem. Thermodyn. 57 (2013) 372–379. https://doi.org/10.1016/j.jct.2012.09.004.

[11] M. Deetlefs, K.R. Seddon, M. Shara, Predicting physical properties of ionic liquids, Phys. Chem. Chem. Phys. 8 (2006) 642–649. https://doi.org/10.1039/b513453f.

[12] J.L. Viesca, P. Oulego, R. González, H. Guo, A.H. Battez, P. Iglesias, Miscibility, corrosion and environmental properties of six hexanoate- and sulfonate-based protic ionic liquids, J. Mol. Liq. 322 (2021). https://doi.org/10.1016/j.molliq.2020.114561.

[13] H. Guo, T.W. Smith, P. Iglesias, The study of hexanoate-based protic ionic liquids used as lubricants in steel-steel contact, J. Mol. Liq. 299 (2020) 112208. https://doi.org/10.1016/j.molliq.2019.112208.

[14] M.R.O. Vega, K. Parise, L.B. Ramos, U. Boff, S. Mattedi, L. Schaeffer, C.F. Malfatti, Protic

ionic liquids used as metal-forming green lubricants for aluminum: Effect of anion chain length, Mater. Res. 20 (2017) 675–687. https://doi.org/10.1590/1980-5373-MR-2016-0626.

[15] A. Tzani, M. Elmaloglou, C. Kyriazis, D. Aravopoulou, I. Kleidas, A. Papadopoulos, E. Ioannou, A. Kyritsis, E. Voutsas, A. Detsi, Synthesis and structure-properties relationship studies of biodegradable hydroxylammonium-based protic ionic liquids, J. Mol. Liq. 224 (2016) 366–376. https://doi.org/10.1016/j.molliq.2016.09.086.

[16] P. Hu, D. Ph, Z. Wu, J. Wang, Y. Huang, Q. Liu, S. Zhou, Corrosion inhibiting performance and mechanism of protic ionic liquids as green brass inhibitors in nitric acid, Green Energy Environ. (2019). https://doi.org/10.1016/j.gee.2019.11.003.

[17] O. Olivares-Xometl, I. V. Lijanova, N. V. Likhanova, P. Arellanes-Lozada, H. Hernández-Cocoletzi, J. Arriola-Morales, Theoretical and experimental study of the anion carboxylate in quaternary-ammonium-derived ionic liquids for inhibiting the corrosion of API X60 steel in 1 M H2SO4, J. Mol. Liq. 318 (2020) 114075. https://doi.org/10.1016/j.molliq.2020.114075.

[18] X. Gao, Y. Wu, Q. Huang, Y. Jiang, D. Ma, T. Ren, The inhibition behavior of novel ionic liquids for magnesium alloy in NaCl solution: Experimental and theoretical investigation, J. Mol. Liq. 324 (2021) 114732. https://doi.org/10.1016/j.molliq.2020.114732.

[19] Y. Song, Y. Xia, Z. Liu, Influence of cation structure on physicochemical and antiwear properties of hydroxyl-functionalized imidazolium bis(trifluoromethylsulfonyl)imide ionic liquids, Tribol. Trans. 55 (2012) 738–746. https://doi.org/10.1080/10402004.2012.701000.

[20] C. Maton, N. De Vos, C. V. Stevens, Ionic liquid thermal stabilities: Decomposition mechanisms and analysis tools, Chem. Soc. Rev. 42 (2013) 5963–5977. https://doi.org/10.1039/c3cs60071h.

[21] Y. Hao, J. Peng, S. Hu, J. Li, M. Zhai, Thermal decomposition of allyl-imidazolium-based ionic liquid studied by TGA-MS analysis and DFT calculations, Thermochim. Acta. 501 (2010) 78–83. https://doi.org/10.1016/j.tca.2010.01.013.

[22] Y. Cao, T. Mu, Comprehensive investigation on the thermal stability of 66 ionic liquids by thermogravimetric analysis, Ind. Eng. Chem. Res. 53 (2014) 8651–8664. https://doi.org/10.1021/ie5009597.
